# White-gutted soldiers: simplification of the digestive tube for a non-particulate diet in higher Old World termites (Isoptera: Termitidae)

**DOI:** 10.1007/s00040-017-0572-9

**Published:** 2017-07-12

**Authors:** R. H. Scheffrahn, T. Bourguignon, C. Bordereau, R. A. Hernandez-Aguilar, V. M. Oelze, P. Dieguez, J. Šobotnik, A. Pascual-Garrido

**Affiliations:** 1 Fort Lauderdale Research and Education Center, University of Florida, 3205 College Avenue, Davie, FL 33314 USA; 2 Okinawa Institute of Science and Technology Graduate University, 1919-1 Tancha, Onna-son, Okinawa, 904-0495 Japan; 3 UMR CNRS 5548, Communication Chimique, Université de Bourgogne Développement, 21000 Dijon, France; 4 Department of Biosciences, Centre for Ecological and Evolutionary Synthesis (CEES), University of Oslo, P.O. Box 1066, Blindern, 0316 Oslo, Norway; 5 Department of Primatology, Max Planck Institute for Evolutionary Anthropology, Deutscher Platz 6, 04103 Leipzig, Germany; 6 Faculty of Forestry and Wood Sciences, Czech University of Life Sciences, Prague, Czech Republic; 7 Leverhulme Trust Early Career Fellow, School of Archaeology, University of Oxford, Oxford, OX1 3QY UK

**Keywords:** Apicotermitinae, Cubitermitinae, Foraminitermitinae, Macrotermitinae, Termitinae, Enteric valve armature, Proctodeum

## Abstract

Previous observations have noted that in some species of higher termites the soldier caste lacks pigmented particles in its gut and, instead, is fed worker saliva that imparts a whitish coloration to the abdomen. In order to investigate the occurrence of this trait more thoroughly, we surveyed a broad diversity of termite specimens and taxonomic descriptions from the Old World subfamilies Apicotermitinae, Cubitermitinae, Foraminitermitinae, Macrotermitinae, and Termitinae. We identified 38 genera that have this “white-gutted” soldier (WGS) trait. No termite soldiers from the New World were found to possess a WGS caste. Externally, the WGS is characterized by a uniformly pale abdomen, hyaline gut, and proportionally smaller body-to-head volume ratio compared with their “dark-gutted” soldier (DGS) counterparts found in most termitid genera. The WGS is a fully formed soldier that, unlike soldiers in other higher termite taxa, has a small, narrow, and decompartmentalized digestive tube that lacks particulate food contents. The presumed saliva-nourished WGS have various forms of simplified gut morphologies that have evolved at least six times within the higher termites.

**Electronic supplementary material:**

The online version of this article (doi:10.1007/s00040-017-0572-9) contains supplementary material, which is available to authorized users.

## Introduction

Trophallaxis is the mouth-to-mouth (stomodeal) or anus-to-mouth (proctodeal) exchange of alimentary contents between members of a social insect colony (Wilson 1971). Termite soldiers lack chewing mandibles and must obtain their food as liquids or pastes offered by nestmate workers (Grassé 1949). In the lower wood-feeding termites (e.g., Kalotermitidae and Rhinotermitidae), soldiers are fed with worker gut contents which consist of mixtures of masticated wood particles and saliva (Grassé 1982; Noirot 1969). The diets of most higher termites (Termitidae) are much more varied (Donovan et al. 2001), and soldiers are nourished by stomodeal trophallaxis with particles of worker-masticated wood, leaf litter, soil, fungus, bacteria, herbaceous plants, lichens, algae, or some derivation of these (e.g., sound vs. severely decayed wood). Workers of soil-feeding termites, which constitute the majority of termitid genera, ingest masticated mixtures of humus, roots, mycelia, lignified tissue, silica grains, etc. (Sleaford et al. 1996; Donovan et al. 2001; Donovan 2002) which are transferred to their soldiers. These particulate suspensions impart a contrastingly darker coloration to the gut tubes of both mature workers and soldiers, which are visible through their opaque or nearly transparent integument.

Grassé (1949) reported that in some higher termites (probably mostly in soil-feeding taxa), the diet of soldiers may consist wholly of the worker’s saliva in its clear or opalescent form. Noirot (1955) observed that soldiers of *Procubitermes curvatus* Silvestri (Cubitermitinae) have an exclusively liquid diet and their digestive tube, contrary to that of their workers, lacks particulate food content. Noirot (1955) further noted that soldiers of *Pericapritermes urgens* Silvestri (Termitinae) also have an exclusively liquid diet which imparts a whitish-yellowish color to their abdomens. Noirot and Noirot-Timothée (1969) reported that soldiers of *Basidentitermes, Fastigitermes, Orthotermes, Proboscitermes, Procubitermes,* and *Promirotermes* also have a strict liquid (salivary) diet but did not mention their abdominal coloration. Finally, in her key of southern African termitine genera, Uys (2002) used abdominal coloration, an artifact of a salivary diet, in her first couplet (“creamy yellow to creamy white” for the African genera *Angulitermes, Basidentitermes, Lepidotermes, Noditermes, Pericapritermes, Promirotermes, Unguitermes,* and *Unicornitermes*; and “greyish-black” for *Amitermes, Batillitermes, Crenetermes, Cubitermes, Euchilotermes, Microcerotermes, Okavangotermes, Ovambotermes,* and *Termes*).

Despite its importance, the morphological and phylogenetic underpinnings of this bipartite soldier condition have received little or no additional attention. This led us to examine soldiers from an extensive collection of termites worldwide and to interpret soldier description literature. We show that Old World termitid soldiers can be sorted into two groups: the “white-gutted” soldier group (WGS) and the more common “dark-gutted” soldier group (DGS). Herein, we identify WGS genera, compare the external and internal morphology of WGS and DGS from selected genera of higher termites, and present information from the literature that we use to reconstruct the ancestral state of soldier gut type from a recently published termite phylogenetic tree.

## Materials and methods

Photos of whole or partial termite bodies were taken as multi-layer montages using a Leica M205C stereomicroscope controlled by Leica Application Suite version 3 software. Preserved specimens were taken from 85% ethanol and suspended in a pool of Purell^®^ Hand Sanitizer (70% ethanol) to position the specimens within a clear plastic Petri dish. Enteric valve armature (EVA) images were taken from slide mounts using a Leica CTR 5500 compound microscope with differential interference contrast optics and the same montage software. All photographed specimens and those listed in Table S1 (electronic supplementary material) are housed in the University of Florida Termite Collection in Davie, Florida, which contains 42,595 colony samples of 229 described and new genera. African termite specimens were collected in the field for primate nutrition and/or termite diversity studies between 1989 and 2016. Terminology of the worker gut follows that of Sands (1998) and Noirot (2001).

We used one phylogenetic tree of Termitidae recently published by Bourguignon et al. (2017) to reconstruct the ancestral state of the soldier gut. We pruned the tree so that one representative of each genus for which we know the type of soldier gut remains. The tree was a Bayesian phylogenetic chronogram inferred from full mitochondrial genomes with third codon position excluded. Two states were considered, WGS and DGS. We used the function “ace” of the package phytools (Revell 2012) implemented in R version 3.2.0. The model implemented by ace was a maximum likelihood model with equal rate of transition between states.

## Results

**Fig. 1:**
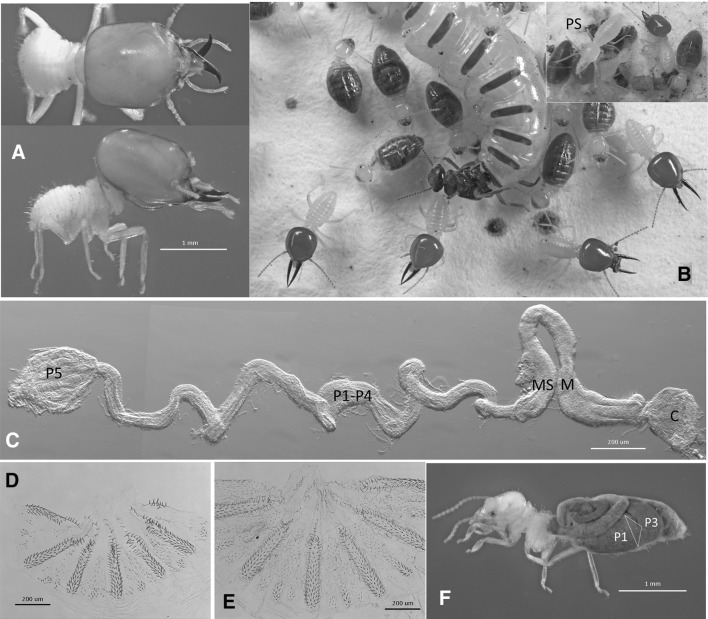
**a** White-gutted soldier (WGS) of *Basidentitermes* n. sp.; **b** *B. malelaensis* Emerson live habitus of soldiers, workers, and queen (inset presoldier (PS) and nestmates of *B*. n. sp.; **c** uncoiled gut of *B*. n. sp. WGS; **d** enteric valve armature (EVA) of *B*. n. sp. worker; **e** EVA of *B. malelaensis* worker; and **f** lateral view of *B. malelaensis* worker with abdominal integument removed (*triangle marks* location of EVA). *C* crop (includes gizzard), *M* mesenteron, *MS* mixed segment, P1–P5 first through fifth proctodeal segments

Our examination of termite specimens (Table S1), and the descriptive wording of taxa we lacked revealed that at least 38 Old World genera have the WGS morph (Table 1). All are soil/humus feeding species (non-flocculent and silica particles abundant in worker gut) with the exception of the plant-feeding *Angulitermes* (Debelo and Degaga 2014), *Eremotermes* (Akhtar and Sarwar 2003), *Forficulitermes* (Scheffrahn and Křeček 2015), *Promirotermes* (Davies et al. 2015), *Synhamitermes* (Shanbhag and Sundararaj 2012), and the fungus feeding *Acanthotermes*, *Pseudacanthotermes*, and *Synacanthotermes*, that have abundant flocculent contents in the worker gut but lack silica particles. These eight genera all lack soldiers with asymmetrical snapping mandibles (Table 1). The WGS morph is recognizable externally by its uniformly pale abdomen and proportionally smaller body-to-head volume ratio (Figs. S1, S2) compared with the darker abdomens and larger body-to-head proportions of the DGS taxa (Fig. S3). Unlike Noirot’s 1969 “white soldier” or the equivalent term “presoldier” (Noirot and Pasteels 1987) which describe the stage before the final soldier molt (Fig. 1b inset), the WGS are fully developed and possess functional mandibles.

**Table 1:** White-gutted soldier genera including subfamily, regional distribution (total no. species), mandible type, abdomen/body coloration as given in reference

Genus	Subfamily	Region^a^, no. species	Mandible	Coloration	References
*Acanthotermes*	Macrotermitinae	Eth, 1	Piercing, cutting	White^b^	Current paper
*Angulitermes*	Termitinae	Eth, Ore, Pale, 29	Symmetrical snap	Creamy white	Uys (2002), Harris (1964)
*Basidentitermes*	Cubitermitinae	Eth, 8	Piercing, cutting	White	Current paper
*Captritermes*	Termitinae	Eth, 1	Asymmetrical snap	White	Current paper
*Dicuspiditermes*	Termitinae	Ore, 20	Asymmetrical snap	Yellowish white	Akhtar (1975)
*Eremotermes*	Termitinae	Eth, Ore, Pale, 10	Piercing, cutting	Whitish	Chhotani (1997)
*Eburnitermes*	Apicotermitinae	Eth, 1	Piercing, cutting	Yellowish white	Noirot (1966)
*Euhamitermes*	Apicotermitinae	Ore, 24	Crushing	Body lighter than head	Chhotani (1975)
*Eurytermes*	Apicotermitinae	Ore, 6	Crushing	Whitish	Chhotani (1997)
*Fastigitermes*	Cubitermitinae	Eth, 1	Piercing, cutting	White	Current paper
*Forficulitermes*	Termitinae	Eth, 1	Piercing, cutting	Abdomen paler than head	Emerson (1960), Scheffrahn and Křeček (2015)
*Homallotermes*	Termitinae	Ore 4	Asymmetrical snap	Whitish	Chhotani (1997)
*Indocapritermes*	Termitinae	Ore, 1	Asymmetrical snap	Whitish	Chhotani (1997)
*Indotermes*	Apicotermitinae	Ore, 10	Piercing, cutting	White	Current paper
*Krishnacapritermes*	Termitinae	Ore, 2	Asymmetrical snap	Whitish	Chhotani (1997)
*Labiocapritermes*	Termitinae	Ore, 1	Asymmetrical snap	Whitish	Chhotani (1997)
*Labritermes*	Foraminitermitinae	Ore, 3	Piercing, cutting	Yellowish white	Anonymous^c^
*Lepidotermes*	Cubitermitinae	Eth, 9	Piercing, cutting	White	Uys (2002)
*Mirocapritermes*	Termitinae	Ore, 8	Asymmetrical snap	Yellowish white	Chhotani (1997)
*Mucrotermes*	Cubitermitinae	Eth, 2	Piercing, cutting	Abdomen paler than pronotum	Emerson (1960)
*Noditermes*	Cubitermitinae	Eth, 7	Piercing, cutting	White	Current paper
*Orthotermes*	Cubitermitinae	Eth, 2	Piercing, cutting	White	Current paper
*Pericapritermes*	Termitinae	Eth, Ore, Pale, Pap, 40	Asymmetrical snap	White	Current paper
*Pilotermes*	Cubitermitinae	Eth, 1	Piercing, cutting	Abdomen whitish	Emerson (1960)
*Proboscitermes*	Cubitermitinae	Eth, 2	Piercing, cutting	Hyaline	Scheffrahn and O’Malley (2010)
*Procapritermes*	Termitinae	Ore, 13	Asymmetrical snap	Pale yellow	Thapa (1982)
*Procubitermes*	Cubitermitinae	Eth, 9	Piercing, cutting	White	Current paper
*Profastigitermes*	Cubitermitinae	Eth, 1	Piercing, cutting	Abdomen paler than pronotum	Emerson (1960)
*Promirotermes*	Termitinae	Eth, 10	Symmetrical snap	White	Current paper
*Pseudacanthotermes*	Macrotermitinae	Eth, 6	Piercing, cutting	White^b^	Current paper
*Pseudocapritermes*	Termitinae	Ore, 2	Asymmetrical snap	Creamy white	Chhotani (1997)
*Quasitermes*	Termitinae	Eth, 1	Asymmetrical snap	Pale	Katie Cribbs
*Sinocapritermes*	Termitinae	Ore, 16	Asymmetrical snap	Abdomen without soil content	Chiu et al. (2015)
*Speculitermes*	Apicotermitinae	Ore, 12	Crushing	White	Chhotani (1997)
*Synacanthotermes*	Macrotermitinae	Eth, 3	Piercing, cutting	White	Current paper
*Synhamitermes*	Termitinae	Ore, 4	Piercing, cutting	Yellow white	Chhotani (1997)
*Unguitermes*	Cubitermitinae	Eth, 7	Piercing, cutting	Creamy white	Uys (2002)
*Unicornitermes*	Cubitermitinae	Eth, 1	Piercing, cutting	Creamy white	Uys (2002)

^a^ *Eth* Ethiopian, *Ore* oriental, *Pale* palarctic, *Pap* papuan

^b^ Underlying dark cuticle

^c^ http://termitesandants.blogspot.com/2012/05/labritermes.html

**Fig. 2:**
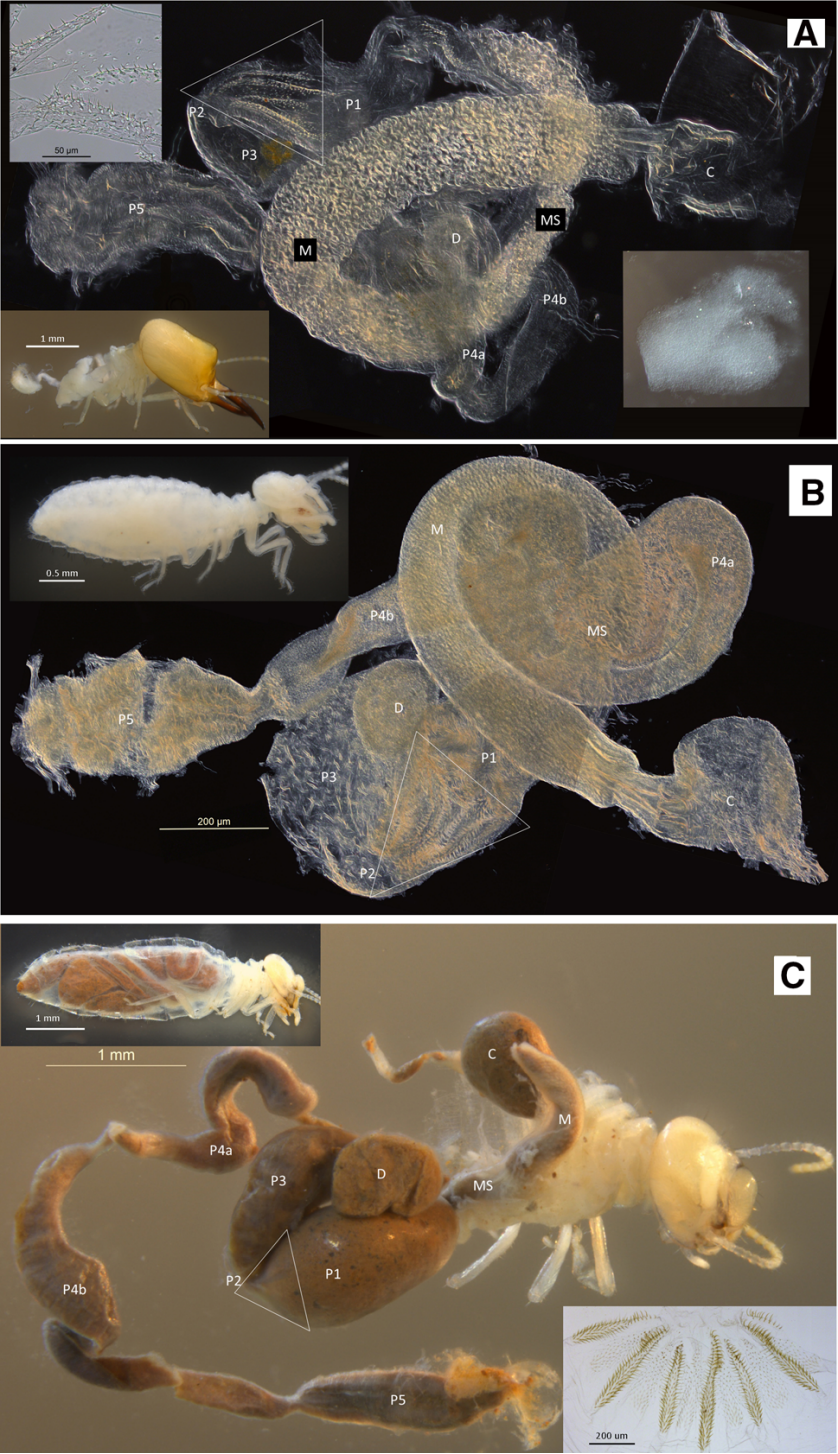
*Procubitermes* sp. **a** White-gutted soldier (WGS) whole gut (insets *upper left* is half of soldier enteric valve armature (EVA), *lower left* is WGS with abdominal integument removed, and *right inset* is the contents of the WGS crop); **b** unwound gut of last instar larva (LIL) (*top inset* whole LIL); **c** worker with gut unwound (*top inset* whole worker; *bottom inset* worker EVA). *D* diverticulum. See Fig. 1 for abbreviation definitions

**Fig. 3:**
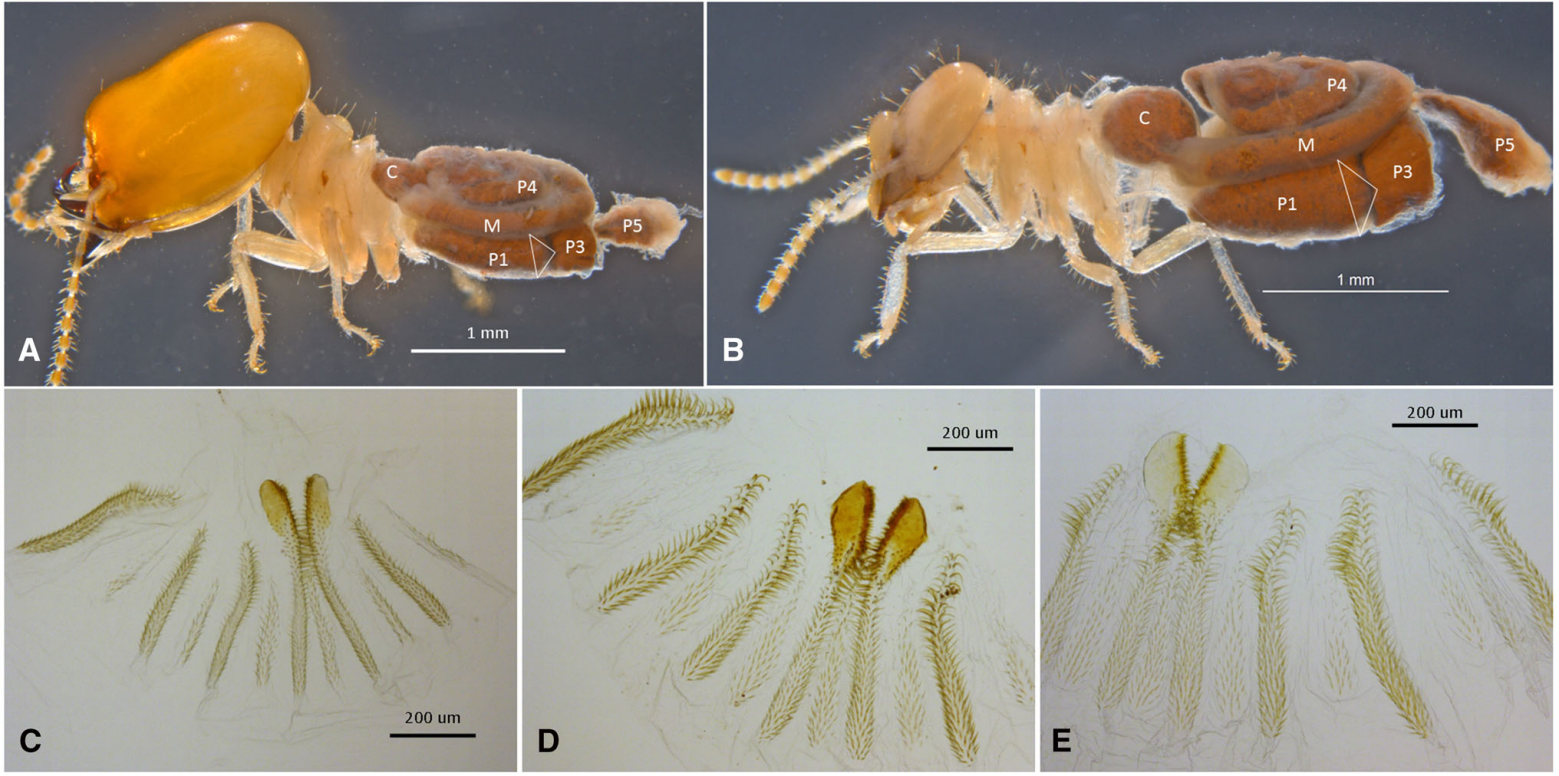
*Cubitermes schereri* (Rosen) **a** Dark-gutted soldier (DGS); **B** worker of with abdominal integument removed. Enteric valve armature of **c** DGS, **d** worker, and **e** last instar larva. See Fig. 1 for abbreviation definitions

Internally, the digestive tubes of WGS are variously simplified, shortened, narrowed, and/or decompartmentalized, and lack particulate food contents (Figs. 1, 2, S4) present in the workers of their species (Figs. 1b, f, 2c) or in soldiers and workers of DGS species (Figs. 3, S3). Several gut morphologies are represented in the WGS group. In *Basidentitermes*, the WGS gut segments form a very long tube with an enlarged crop (C) and rectum (P5) at either end (Fig. 1c). The midgut (mesenteron, M, and mixed segment, MS), can barely be differentiated because they differ from each other only slightly in shape and diameter. The proctodeal segments P1–P4 of the *Basidentitermes* WGS show no discernable junctures and the EVA is absent. In the *Promirotermes* WGS (Fig. S4a), the crop, mesenteron, mixed segment, and rectum are all well developed but the proctodeal segments P1–P4 form a long and serpentine tube without clear sectional delineations. The EVA is absent in the *Promirotermes* soldier. The *Pericapritermes* WGS (Fig. S4b) has a rather short gut tube and all segments are recognizable but it too lacks the EVA. Unlike the previous genera, the gut segments of the *Procubitermes* WGS are all recognizable and it has remnants of the enteric valve armature (Fig. 2a), albeit less developed than that of the LIL (Fig. 2b) or that of the fully sclerotized EVA of the fully formed worker (Fig. 2c). In comparison, the DGS taxa have more robust hindgut morphologies similar to their workers, and, akin to workers, the entire DGS alimentary tract contains grainy particulate matter (e.g., *Cubitermes schereri* Rosen, Fig. 3a, b). Additionally, the DGS soldier has a fully developed EVA similar to that of its worker and LIL (Fig. 3c–e).

**Fig. 4:**
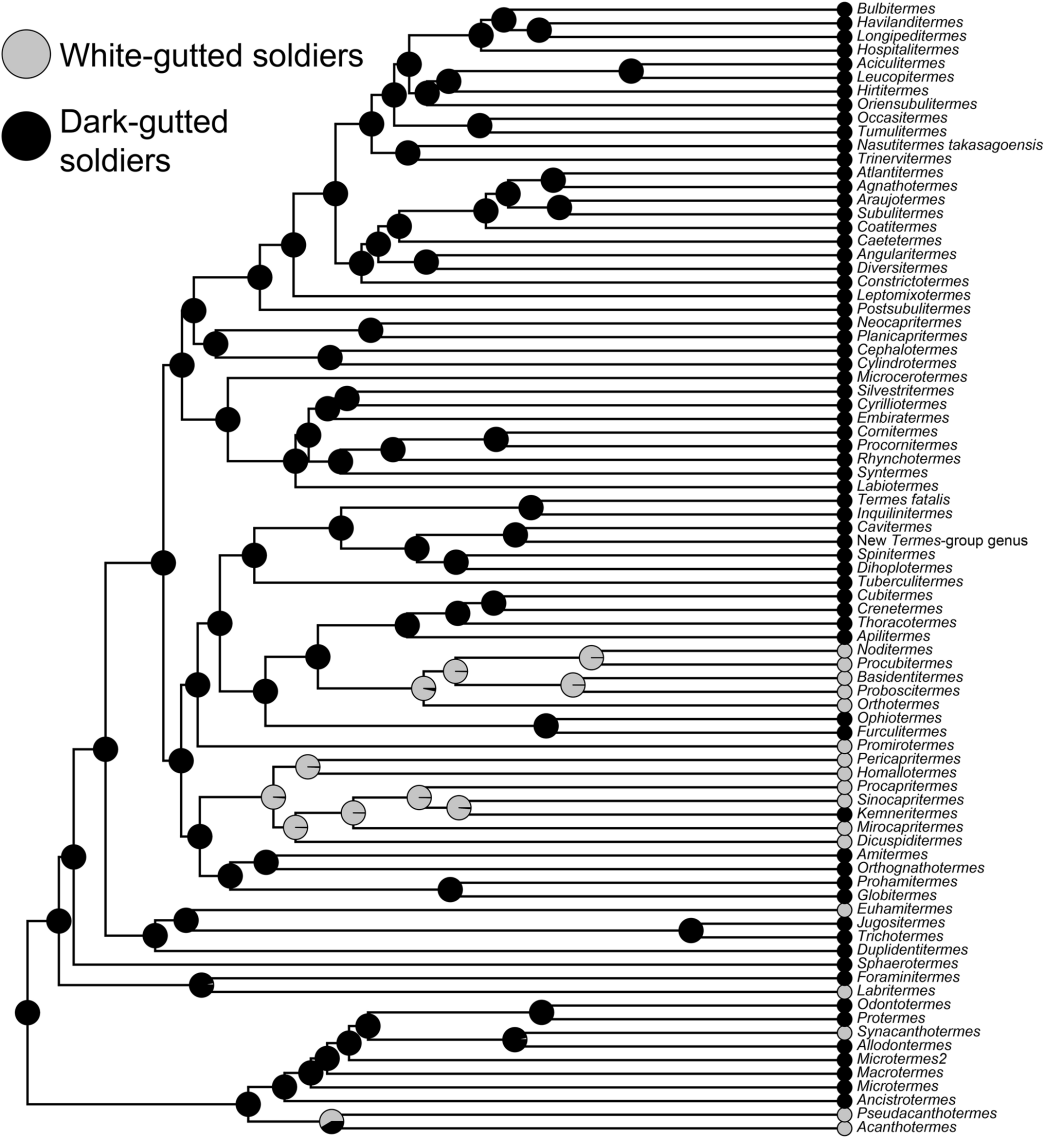
Phylogenetic tree of Termitidae reproduced from Bourguignon et al. (in press). Node pie charts show the probabilities for ancestral states to be white-gutted and dark-gutted soldiers. The ancestral states were reconstructed using a Likelihood model with equal rate of transition between states

Our phylogenetic tree includes 83 taxa belonging to 82 genera (Fig. 4). The ancestral state reconstruction shows, with strong support, that WGS evolved at least once in Cubitermitinae, twice in Termitinae, and once in Apicotermitinae. We also included some members of the basal Termitidae subfamilies, i.e., Foraminitermitinae, Macrotermitinae, and Sphaerotermitinae and found that WGS also evolved in these subfamilies: once in the soil-feeding *Labritermes* (Foraminitermitinae), and at least twice in Macrotermitinae.

## Discussion

13 WGS genera of Apicotermitinae, Cubitermitinae, Macrotermitinae, and Termitinae are included in both Table 1 and the phylogenetic tree we used to reconstruct the ancestral state of soldier gut. Our tree shows that WGS evolved from DGS at least four times in these lineages. Because our tree did not include all the species recorded with WGS, there is a possibility that WGS evolved independently in more lineages. One of them is *Forficulitermes*, a genus recently transferred from Cubitermitinae to Termitinae (Scheffrahn and Křeček 2015). The absence of WGS taxa in the New World is enigmatic given the common ancestry of both New and Old World Apicotermitinae and Termitinae (Bourguignon et al. 2017).

In Noirot’s 1966 description of *Eburnitermes grassei* (Apicotermitidae), he reports that soldiers of this species possess a “yellowish-white abdomen due to the fact that the digestive tract does not contain solid food”. Due to lack of material, we did not examine the gut contents of the *E. grassei* soldier; however, other apicotermitine genera with soldiers examined including *Allognathotermes* sp., *Coxotermes boukokoensis* Grassé and Noirot, *Duplidentitermes* sp., *Heimitermes* sp., *Jugositermes tuberculatus* Emerson, *Phoxotermes cerberus* Collins, and *Rostrotermes cornutus* Grassé all possess the DGS form and contain solid dark particulates in their guts.

For the Termitidae, salivary secretions from workers are the sole or primary nutrients for all immature stages (Grassé 1982; Noirot 1969). The immature stages, white in color, are generally found near reproductive centers of the nest for all termite feeding groups, including soil-feeders (Fig. S5a–d) and wood-feeders (Fig. S5e, f). Casual observations of the guts of the last instar larvae (LIL, usually the second instar, Noirot 1969) and presoldier stages sampled from about 120 termitid genera, both Old and New World, show no traces of particulate content (Scheffrahn, pers. obs.).

Although trophallaxis and salivary secretions in the WGS have not been studied, some inference from the DGS group may be useful in understanding the diet common to all dependent castes in the Termitidae. For example, using radionuclide tracing, Alibert (1963) confirmed that larvae and presoldiers of *C. fungifaber* Sjöstedt received salivary secretions from workers, while mature soldiers and workers received regurgitated foregut contents. Studies of New World Termitidae show that worker salivary (labial) gland secretions contain complex aqueous admixtures of proteins and other non-volatile compounds (Sillam-Dussès et al. 2012) which nourish dependent castes. Billen et al. (1989) found that the worker salivary glands of *Macrotermes bellicosus* (Smeathman) are substantial and consist of three secretory cell types, while the soldier gland has only a single type that secretes a defense fluid (Prestwich 1979). However, upon reaching maturity, the DGS guts are filled with dark particulate matter from stomodeal trophallaxis (e.g., Figure 3a). Therefore, the worker-to-larval + presoldier trophallaxis in DGS and the worker-to-larval + presoldier + soldier trophallaxis in the WGS is a special form of nutrient sharing with food originating from the salivary gland (buccal trophallaxis?) and not from the foregut (stomodeal trophallaxis).

For the first time, this study introduces the external and internal morphology of the WGS underlying the observations of Noirot (1955, 1966), Noirot and Noirot-Timothée (1969), and Grassé (1949, 1982). Although we did not analyze the WGS worker saliva or guts, the matter found in WGS soldier crops (Fig. 2a inset) has the appearance of a secretory substance. Although obvious in both live and preserved material, the coloration of the WGS is omitted in taxonomic descriptions by some renowned taxonomists such as Emerson AE, Krishna K, and Sands WA. We suspect that their focus was on the complex external morphology of the soldier head capsule, and that the internal anatomy of soldiers had been supplanted by the greater interest in worker internal anatomy.

Compared with other insects, the digestive tube of higher termite workers is morphologically complex (Noirot 2001) and provides for multifaceted nutrient metabolism (Bignell 2010). Although the soldier gut in all taxa is largely overlooked, the similarity of the DGS to its workers (e.g., Fig.  3c, d) suggests similar structure and function. The relatively well-developed midgut (M + MS) in WGS (Figs. 1c, 2a, and S4) is similar to DGS and suggests that its proteolytic function (Ji and Brune 2005) is retained as might be expected from a salivary diet. The termitid worker gut has evolved to optimize microbial symbiosis (Brune 2013). In soil-feeding termites, the gut digests particulates which are comparably nutrient poor and recalcitrant. To accomplish this, soil-feeding termites have compartmentalized physiochemical gut environments to nurture their prokaryotic symbionts (Brune 2013), no doubt at some energy cost. One advantage of the WGS/LIL nutritional scheme over that of the particulate feeders might be to redirect the energy demands lost to soil digestion toward defensive function (WGS) and growth (LIL). For this, the mature workers must carry a greater metabolic burden to feed high-energy secretions to their white-gutted nestmates.

The EVA is well developed in soil-feeding termites. Donovan (2002) and Bignell (2010) suggest that the EVA spines and combs fractionate gut contents by size to expose more digestible organic particles to microbial fermentation. Alternately, Scheffrahn (2013) argues that these structures enhance microbial inoculation of ingested material before entry into the P3. In either case, the EVA probably enhances fermentation of the gut contents before entering the P3 for microbial digestion. As would be expected from a liquid diet, the WGS generally lack an EVA and their P3 is relatively small. Remnants of the EVA and enlargement of the hindgut segments in both the WGS and the LIL of *Procubitermes* (Fig. 2a) suggest that the conversion to a salivary diet is more recent in this genus. We hope that this paper will stimulate studies on the composition of food and dynamics of trophallaxis in all termites.

## Electronic supplementary material

Below is the link to the electronic supplementary material.

Fig. S1 Soldiers of selected cubitermitine genera that exemplify the WGS: **A** *Basidentitermes malelaensis* Emerson, **B** *Orthotermes depressifrons* Silvestri, **C** *Proboscitermes tubuliferus* (Sjöstedt), **D** *Fastigitermes jucundus* (Sjöstedt), **E** *Procubitermes* sp., and **F** *Noditermes wasambaricus* Williams (JPEG 2352 kb)

Fig. S2 Soldiers of selected non-cubitermitine genera that exemplify the WGS: **A** *Promirotermes pygmaeus* Harris, **B** *Pericapritermes* sp. 1, **C** *Pericapritermes urgens* Silvestri, and **D** *Synacanthotermes heterodon* Sjöstedt (JPEG 1439 kb)

Fig. S3 Soldiers of selected cubitermitine genera that exemplify the DGS: **A** *Ophiotermes ugandensis* Fuller (abdominal integument removed in bottom frame), **B** *Apilitermes longiceps* (Sjöstedt), **C** *Cubitermes schereri* (Rosen), **D** *Thoracotermes macrothorax* (Sjöstedt), and **E** *Furculitermes winifredae* Emerson (JPEG 2530 kb)

Fig. S4 White-gutted soldiers with all abdominal tissues remove except the gut: **A** *Promirotermes pygmeus* (inset: detail of gut architecture) and **B** *Pericapritermes* sp. 2. See Fig. 1 for abbreviation definitions (JPEG 838 kb)

Fig. S5 Examples of white brood in the Termitidae (all neotropical): **A** *Ruptitermes* sp., **B** *Silvestritermes* sp., **C** *Labiotermes labralis* (Holmgren), **D** *Anoplotermes banksi* Emerson, **E** *Parvitermes brooksi* (Snyder), and **F** *Microcerotermes arboreus* Emerson (JPEG 4566 kb)

Supplementary material 6 (DOCX 27 kb)
