# Supplementary figures and images for: White-gutted soldiers: simplification of the digestive tube for a non-particulate diet in higher Old World termites (Isoptera: Termitidae)
Source: Insectes Soc. 2017 Jul 12;64(4):525–33. doi: 10.1007/s00040-017-0572-9 (PMC5643368; doi:10.1007/s00040-017-0572-9)

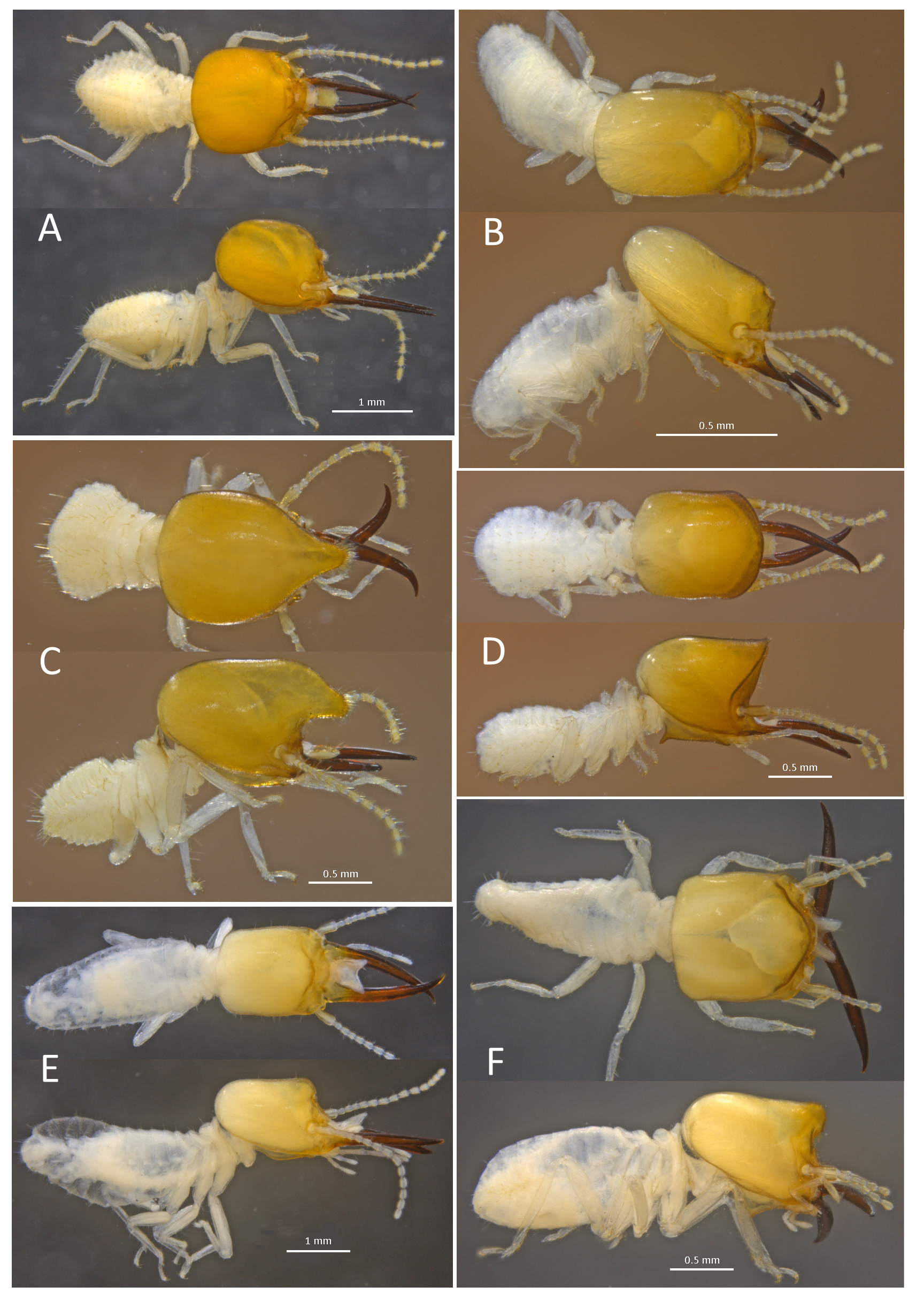

Supplement: Supplementary file 1 — Fig. S1 Soldiers of selected cubitermitine genera that exemplify the WGS: A Basidentitermes malelaensis Emerson, B Orthotermes depressifrons Silvestri, C Proboscitermes tubuliferus (Sjöstedt), D Fastigitermes jucundus (Sjöstedt), E Procubitermes sp., and F Noditermes wasambaricus Williams (JPEG 2352 kb) [file 40_2017_572_MOESM1_ESM.jpg]

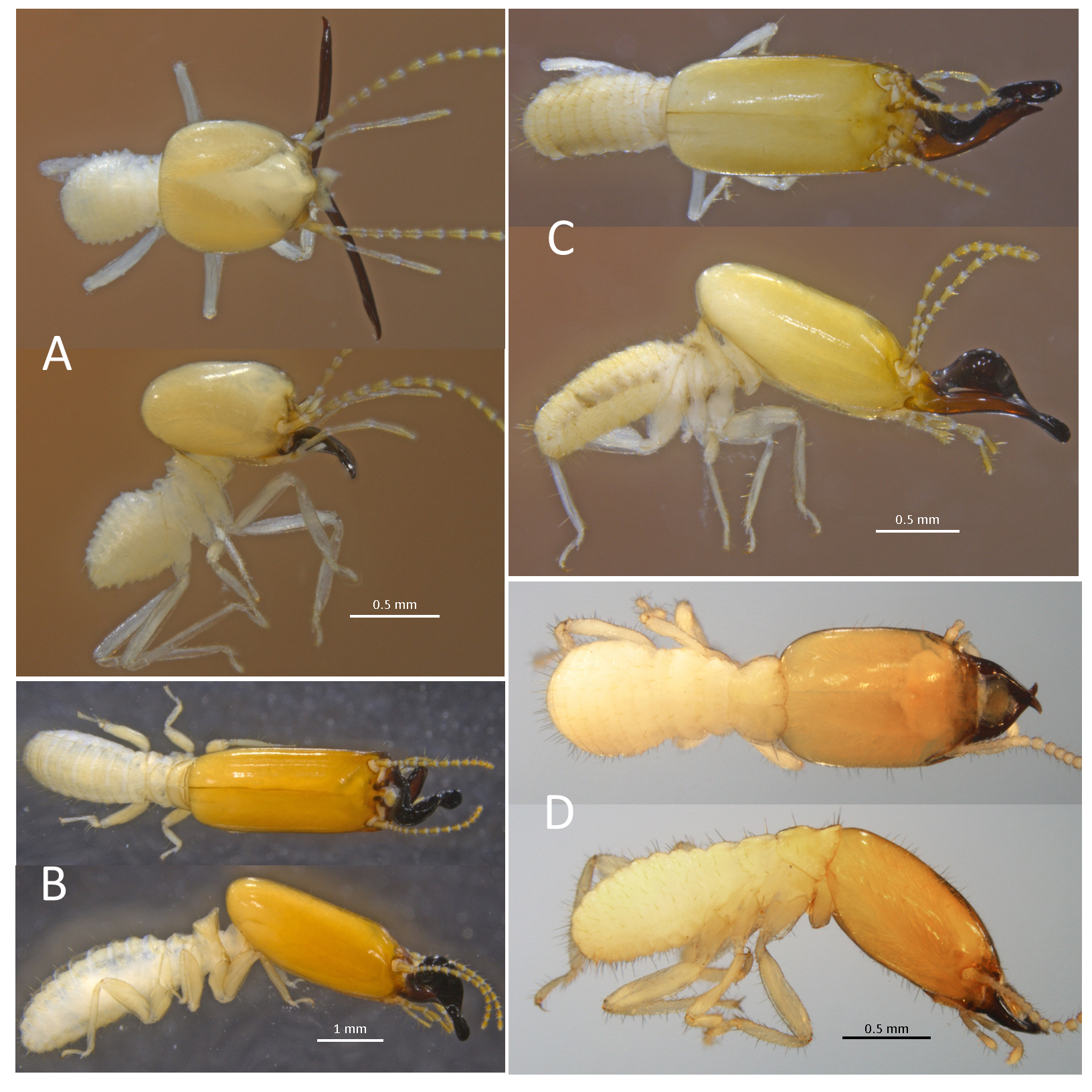

Supplement: Supplementary file 2 — Fig. S2 Soldiers of selected non-cubitermitine genera that exemplify the WGS: A Promirotermes pygmaeus Harris, B Pericapritermes sp. 1, C Pericapritermes urgens Silvestri, and D Synacanthotermes heterodon Sjöstedt (JPEG 1439 kb) [file 40_2017_572_MOESM2_ESM.jpg]

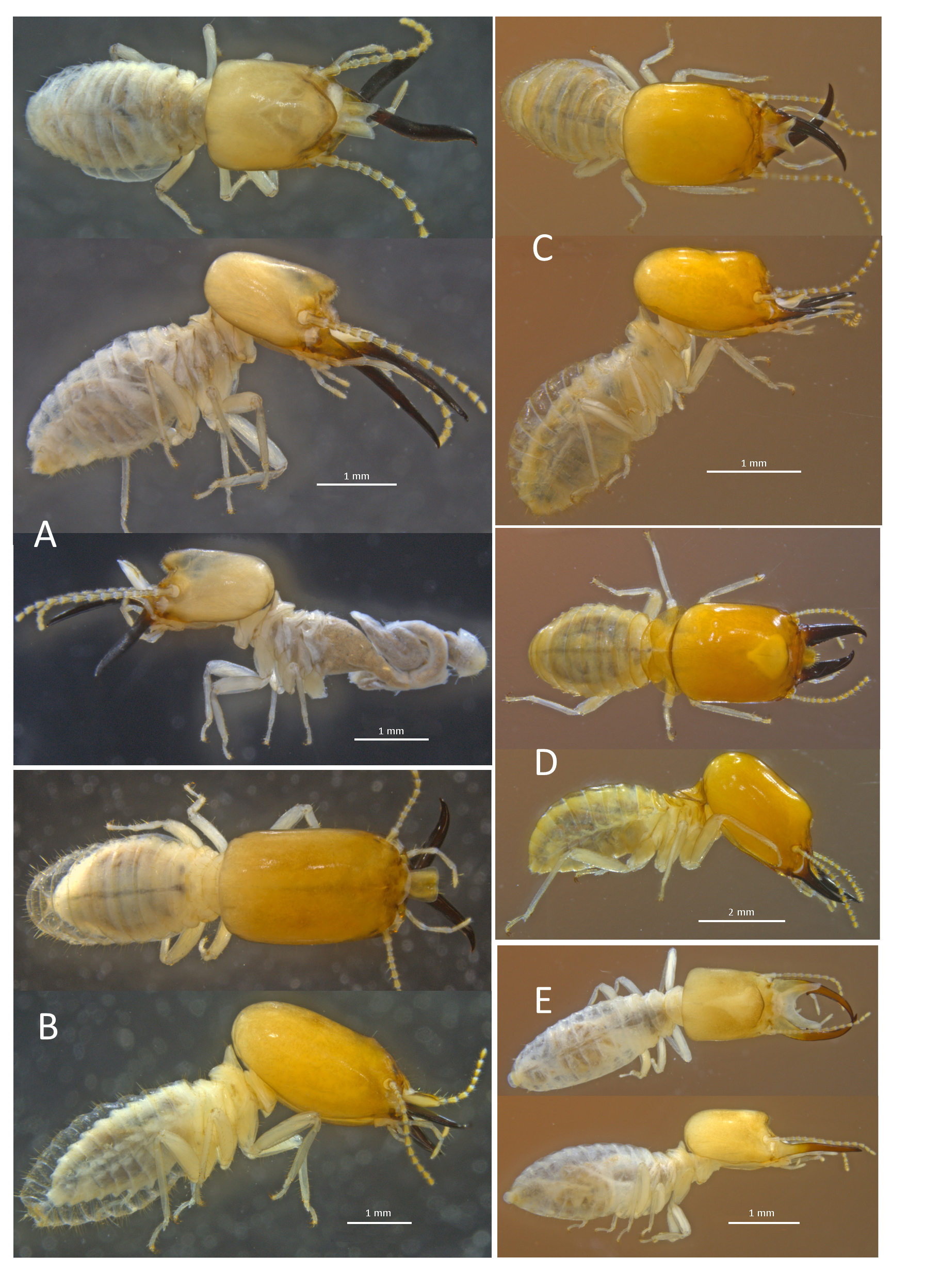

Supplement: Supplementary file 3 — Fig. S3 Soldiers of selected cubitermitine genera that exemplify the DGS: A Ophiotermes ugandensis Fuller (abdominal integument removed in bottom frame), B Apilitermes longiceps (Sjöstedt), C Cubitermes schereri (Rosen), D Thoracotermes macrothorax (Sjöstedt), and E Furculitermes winifredae Emerson (JPEG 2530 kb) [file 40_2017_572_MOESM3_ESM.jpg]

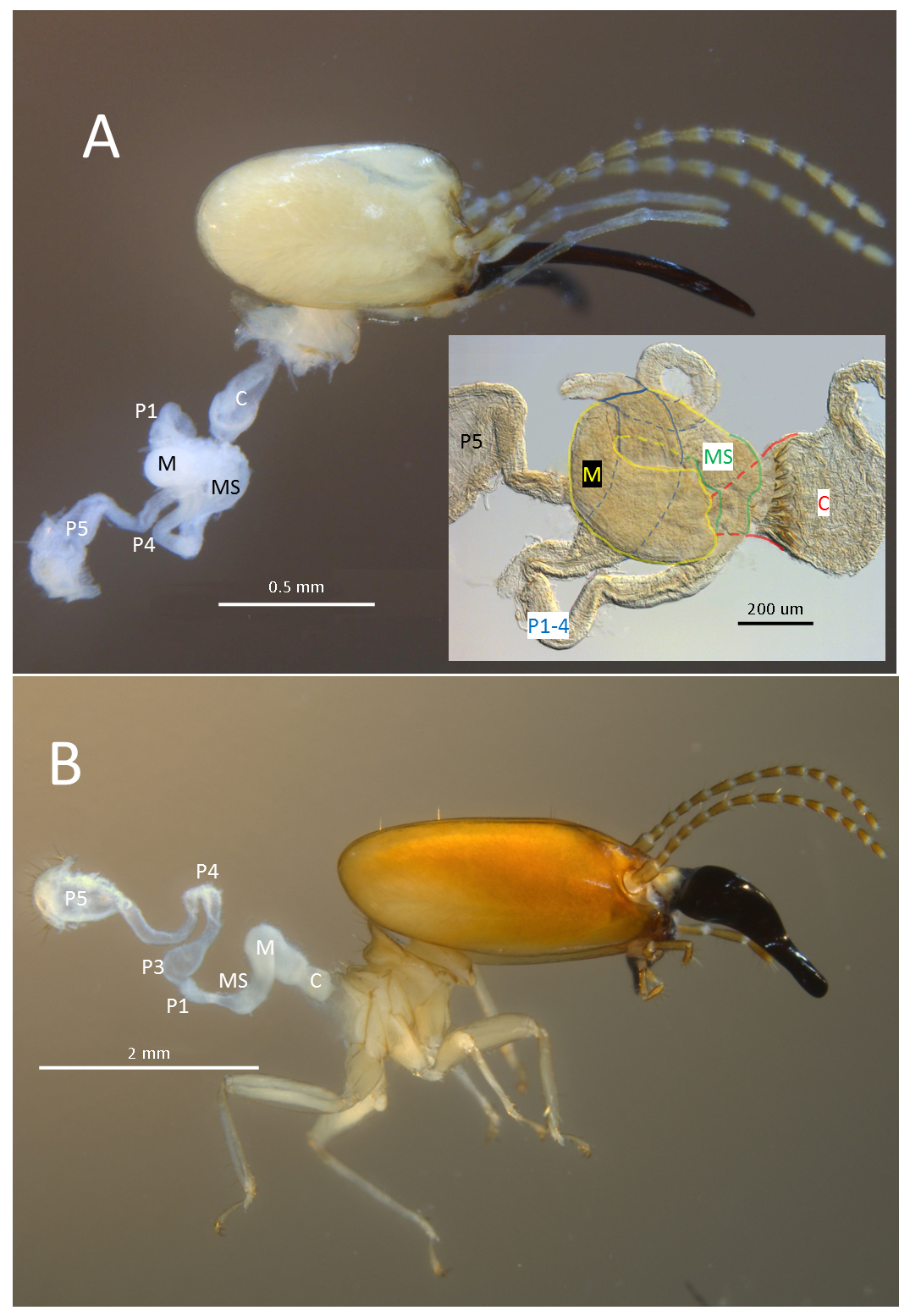

Supplement: Supplementary file 4 — Fig. S4 White-gutted soldiers with all abdominal tissues remove except the gut: A Promirotermes pygmeus (inset: detail of gut architecture) and B Pericapritermes sp. 2. See Fig. 1 for abbreviation definitions (JPEG 838 kb) [file 40_2017_572_MOESM4_ESM.jpg]

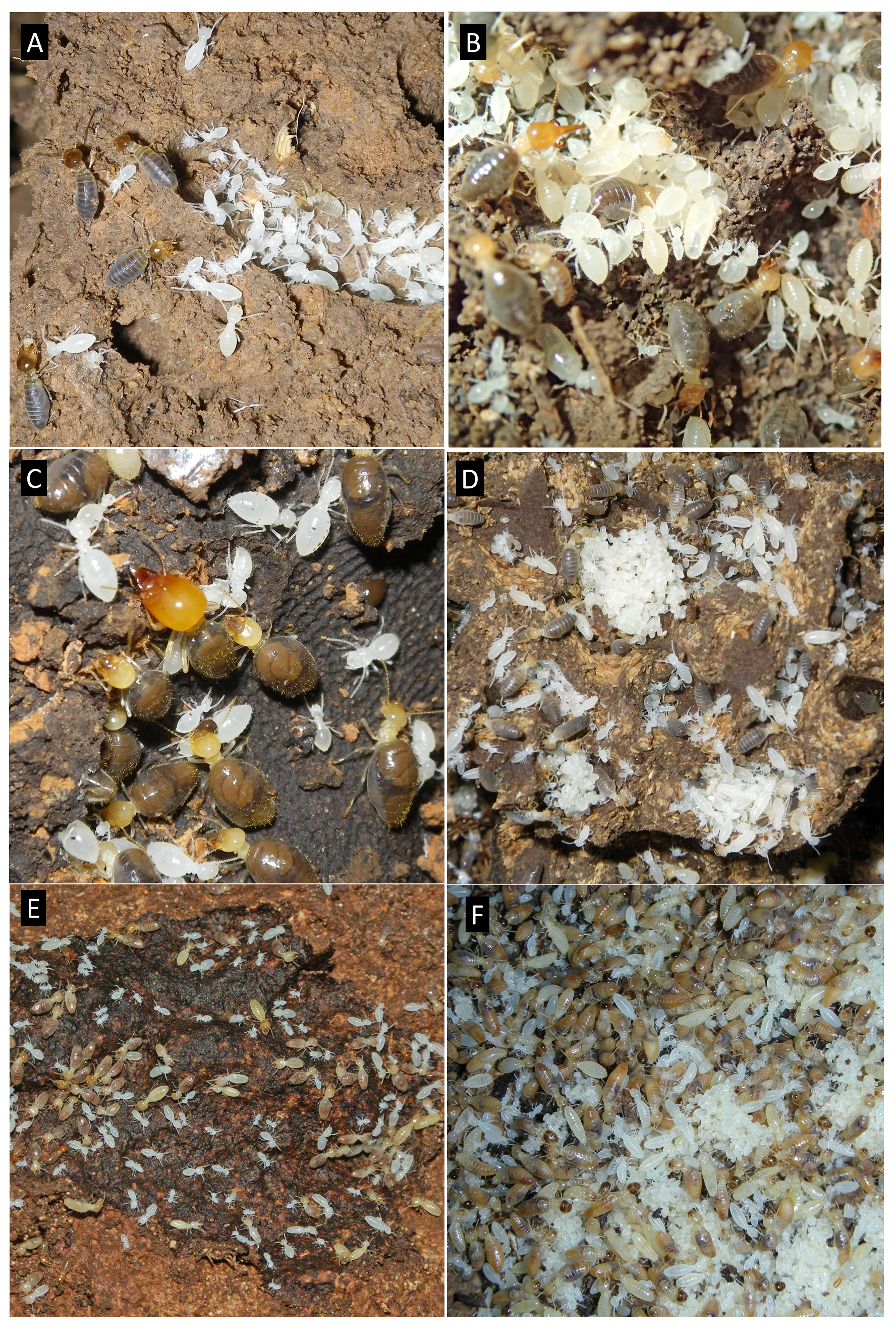

Supplement: Supplementary file 5 — Fig. S5 Examples of white brood in the Termitidae (all neotropical): A Ruptitermes sp., B Silvestritermes sp., C Labiotermes labralis (Holmgren), D Anoplotermes banksi Emerson, E Parvitermes brooksi (Snyder), and F Microcerotermes arboreus Emerson (JPEG 4566 kb) [file 40_2017_572_MOESM5_ESM.jpg]
