# Supplementary material for: White-gutted soldiers: simplification of the digestive tube for a non-particulate diet in higher Old World termites (Isoptera: Termitidae)
Source: Insectes Soc. 2017 Jul 12;64(4):525–33. doi: 10.1007/s00040-017-0572-9 (PMC5643368; doi:10.1007/s00040-017-0572-9)
Supplement: Supplementary file 6 — Supplementary material 6 (DOCX 27 kb) [file 40_2017_572_MOESM6_ESM.docx]

| Table S1. Collection records of Old World termite samples examined in this study. | | | | |  |  |  |
| --- | --- | --- | --- | --- | --- | --- | --- |
| Subfamily | Genus | Species | Ascension No. | Country | Latitude | Longitude | Location |
| White-gutted soldiers | |  |  |  |  |  |  |
| Apicotermitinae | *Euhamitermes* | sp. | ASA335 | China | 21.61 | 101.58 | Wangtianshu |
| Apicotermitinae | *Indotermes* | sp. | ASA339 | China | 22.13 | 100.67 | Mandianhe |
| Cubitermitinae | *Basidentitermes* | *malelaensis* | AFR819 | Tanzania | -4.68 | 29.63 | Gombe Stream N.P. |
| Cubitermitinae | *Basidentitermes* | sp. | AFR2808 | Ivory Coast | 6.20 | -7.71 | South of Tai |
| Cubitermitinae | *Basidentitermes* | n. sp. | AFR295 | Guinea | 7.63 | -8.46 | Nimba Mountains, Tong Bong Bon |
| Cubitermitinae | *Basidentitermes* | n. sp. | AFR205 | Ghana | 6.69 | -1.34 | Bobiri Butterfly Reserve |
| Cubitermitinae | *Fastigitermes* | *jucundus* | AFR1516 | Cameroon | 5.00 | 8.86 | Korup National Park |
| Cubitermitinae | *Forficulitermes* | *planifrons* | AFR1664 | Cameroon | 3.39 | 11.68 | Ebogo, Nyong river |
| Cubitermitinae | *Noditermes* | *wasambaricus* | AFR633 | Tanzania | -4.68 | 29.63 | Gombe Stream National Park |
| Cubitermitinae | *Orthotermes* | *depressifrons* | AFR1702 | Cameroon | 3.39 | 11.68 | Ebogo, Nyong river |
| Cubitermitinae | *Proboscitermes* | *mcgrewi* | AFR1142 | Tanzania | -4.68 | 29.63 | Gombe Stream National Park |
| Cubitermitinae | *Proboscitermes* | *tubuliferus* | AFR1464 | Cameroon | 5.00 | 8.86 | Korup National Park |
| Cubitermitinae | *Procubitermes* | sp. 1 | AFR18 | Guinea | 9.62 | -13.63 | Conakry |
| Cubitermitinae | *Procubitermes* | sp. 2 | AFR422 | Guinea | 7.56 | -8.47 | Nimba Mts. |
| Cubitermitinae | *Procubitermes* | sp. 3 | AFR436 | Guinea | 7.56 | -8.47 | Nimba Mts. |
| Cubitermitinae | *Procubitermes* | sp. 4 | AFR2773 | Ivory Coast | 5.91 | -7.37 | Tai National Park |
| Macrotermitinae | *Acanthotermes* | *acanthothorax* | AFR2807 | Ivory Coast | 6.20 | -7.71 | Tai National Park |
| Macrotermitinae | *Pseudacanthotermes* | *militaris* | AFR1784 | Cameroon | 3.39 | 11.68 | Ebogo |
| Macrotermitinae | *Synacanthotermes* | *heterodon* | AFR1656 | Cameroon | 3.39 | 11.68 | Ebogo, Nyong river |
| Termitinae | *Capritermes* | *capricornis* | AFR2791 | Ivory Coast | 5.79 | -7.32 | Tai National Park |
| Termitinae | *Dicuspiditermes* | *nemorosus* | ASA109 | Malaysia | 5.42 | 100.20 | Penang Is., Pantai Acheh For. Preserve |
| Termitinae | *Pericapritermes* | sp. 2 | AFR1693 | Cameroon | 3.39 | 11.68 | Ebogo, Nyong river |
| Termitinae | *Pericapritermes* | *nitobei* | ASA184 | Taiwan | 24.76 | 121.59 | Fushan |
| Termitinae | *Pericapritermes* | sp. 1 | AFR192 | Ghana | 6.69 | -1.34 | Bobiri Butterfly Reserve |
| Termitinae | *Pericapritermes* | *urgens* | AFR2791 | Ivory Coast | 5.79 | -7.32 | South of Tai |
| Termitinae | *Procapritermes* | sp. | ASA341 | China | 22.13 | 100.67 | Mandianhe |
| Termitinae | *Promirotermes* | *pygmeus* | AFR676 | Tanzania | -4.68 | 29.63 | Gombe Stream National Park |
| Termitinae | *Promirotermes* | *pygmeus* | AFR1056 | Tanzania | -4.68 | 29.63 | Gombe Stream National Park |
| Termitinae | *Pseudocapritermes* | sp. | ASA344 | China | 21.90 | 107.90 | nr. Shiwan Dashan Natl. Park |
| Termitinae | *Sinocapritermes* | *mushae* | ASA148 | Taiwan | 24.77 | 121.74 | Yi-Lan Co. Fu-Shan Research Stn. |
| Dark-gutted soldiers | |  |  |  |  |  |  |
| Apicotermitinae | *Allognathotermes* | sp. | AFR1989 | Nigeria | 6.27 | 5.25 | Okomu N.P. |
| Apicotermitinae | *Coxotermes* | *bonkoensis* | AFR1535 | Cameroon | 3.39 | 11.68 | Ebogo, Nyong river |
| Apicotermitinae | *Duplidentitermes* | *latimentonis* | AFR1718 | Cameroon | 3.39 | 11.68 | Ebogo, Nyong river |
| Apicotermitinae | *Heimitermes* | *laticeps* | AFR1631 | Cameroon | 3.39 | 11.68 | Ebogo, Nyong river |
| Apicotermitinae | *Jugositermes* | *tuberculatus* | AFR1583 | Cameroon | 3.39 | 11.68 | Ebogo, Nyong river |
| Apicotermitinae | *Phoxotermes* | *cerberus* | AFR1388 | Cameroon | 3.39 | 11.47 | Ebogo, Nyong river |
| Apicotermitinae | *Rostrotermes* | *cornutus* | AFR468 | Guinea | 7.56 | -8.47 | Nimba Mts. |
| Cubitermitinae | *Apilitermes* | *longiceps* | AFR1478 | Cameroon | 5.17 | 8.85 | Korup National Park |
| Cubitermitinae | *Apilitermes* | *longiceps* | AFR2329 | Liberia | 6.71 | -9.29 | Bong |
| Cubitermitinae | *Crenetermes* | sp. | AFR2036 | Tanzania | -5.48 | 30.56 | Issa, Ugalla |
| Cubitermitinae | *Cubitermes* | *schereri* | AFR430 | Guinea | 7.56 | -8.47 | Nimba Mts. |
| Cubitermitinae | *Cubitermes* | *schereri* | AFR2789 | Ivory Coast | 5.79 | -7.32 | Tai National Park |
| Cubitermitinae | *Euchilotermes* | cf. *quadriceps* | AFR1951 | Nigeria | 6.27 | 5.25 | Okomu N.P. |
| Cubitermitinae | *Euchilotermes* | sp. | AFR2023 | Tanzania | -5.50 | 30.56 | Issa, Ugalla |
| Cubitermitinae | *Furculitermes* | *winifredae* | AFR1638 | Cameroon | 3.39 | 11.68 | Ebogo, Nyong river |
| Cubitermitinae | *Furculitermes* | sp. | AFR1066 | Tanzania | -4.68 | 29.63 | Gombe Stream N.P. |
| Cubitermitinae | *Okavangotermes* | sp. | AFR1890 | Botswana | -22.35 | 27.22 | 45km N Mahalapye |
| Cubitermitinae | *Ophiotermes* | *grandilabius* | AFR598 | Tanzania | -4.68 | 29.63 | Gombe Stream N.P. |
| Cubitermitinae | *Ophiotermes* | *mirandus* | AFR416 | Guinea | 7.56 | -8.47 | Nimba Mts. |
| Cubitermitinae | *Ophiotermes* | *ugandaensis* | AFR1843 | Kenya | 0.23 | 34.88 | Kalunja Glade, Kakamega Forest |
| Cubitermitinae | *Ophiotermes* | sp. | AFR2003 | Tanzania | -5.49 | 30.57 | Issa, Ugalla |
| Cubitermitinae | *Ovambotermes* | sp. | AFR407 | Guinea | 7.56 | -8.47 | Nimba Mts. |
| Cubitermitinae | *Thoracotermes* | *macrothorax* | AFR438 | Guinea | 7.56 | -8.47 | Nimba Mts. |
| Macrotermitinae | *Macrotermes* | *amplus* | AFR2921 | Gabon | -0.24 | 11.59 | Lopé |
| Macrotermitinae | *Odontotermes* | sp. | AFR713 | Tanzania | -4.68 | 29.63 | Gombe Stream N.P. |
| Sphaerotermitinae | *Sphaerotermes* | *sphaerothorax* | AFR2435 | Nigeria | 7.34 | 11.59 | Gashaka Gumti National Park |
| Termitinae | *Tuberculitermes* | sp. | AFR1264 | Cameroon | 3.39 | 11.47 | Ebogo, Nyong river |
